# Attitudes and perceptions of Nigerians regarding receiving COVID-19 vaccines: an online cross-sectional study

**DOI:** 10.11604/pamj.2022.41.247.33286

**Published:** 2022-03-25

**Authors:** Batholomew Chibuike James, Stephen Sunday Ede, Chinazaekpere Mary Aroh, Chisom Favour Okoh, Chullapant Kanokwan, Mona Lisa Rasip, Wegayehu Enbeyle

**Affiliations:** 1 Public Health Program, Graduate School, Angeles University Foundation, Angeles City, Pampanga, Philippines,; 2 Department of Medical Rehabilitation, College of Medicine, University of Nigeria Enugu, Enugu, Nigeria,; 3 Royal Berkshire National Health Service Foundation Trust, London, United Kingdom,; 4 Department of Medical Rehabilitation, College of Medicine, University of Nigeria Enugu, Enugu, Nigeria,; 5 Endocrinology and Metabolism Unit, Prince Songkhla University Hat-Yai Songkhla Province 90110, Thailand,; 6 National Institutes of Health, Persiaran Setia Murni, Setia Alam, 40170 Shah Alam, Selangor, Malaysia,; 7 College of Health Science, Mizan- Tepi University, Tepi bushira, Ethiopia

**Keywords:** COVID-19, SARS-CoV-2, vaccine, attitudes, perceptions, Nigerians

## Abstract

**Introduction:**

the success of controlling pandemics like COVID-19 can be achieved through its vaccination program. Besides masks, social distance, and good hand hygiene, a rapid vaccine program is crucial in controlling this COVID-19 pandemic. Thus, this study aimed to assess the attitudes and perceptions of Nigerians regarding accepting the COVID-19 vaccine.

**Methods:**

a cross-sectional study was carried out among 334 respondents aged 18 and above from the Southeastern region of Nigeria. A validated questionnaire was used for the data collection through an online Google form. The data analysis was done using SPSS version 25. The association of socio-demographics with attitudes and perceptions was analysed using chi-square tests and Fisher exact tests. At the 95 percent confidence level, a p-value of 0.05 was deemed statistically significant.

**Results:**

sixty point two percent (60.2%) (n = 201) of respondents showed positive attitudes with a mean of (13.96±2.97). Gender was the only demographic factor associated with attitudes (p< 0.001). Respondents with poor perceptions were higher by 53.0% (n = 177) with a mean value of (3.30±1.17). Age, education, gender, and marital status were seen to be associated with perceptions of vaccine acceptance (p<0.05). There was a link between attitudes and perceptions (P> 0.001), as those with positive attitudes also exercised good perceptions.

**Conclusion:**

this study revealed that respondents had positive attitudes regarding COVID-19 vaccination acceptance but negative perceptions of it. As a result, community and health promotion professionals, religious leaders, and local celebrities should use their platforms to raise awareness about the benefits of COVID-19 immunization.

## Introduction

Coronavirus disease is caused by SARS-CoV-2, also known as severe acute respiratory syndrome coronavirus-2 (COVID-19). The pandemic was declared a global hazard in late 2019 [1]. Between January 2020 and November 8^th^ 2021, the WHO recorded 212,765 confirmed cases of COVID-19 in Nigeria, with 2,906 deaths [2, 3]. As of September 25, 2021, 6,552,979 vaccine doses had been given out to a total population of 212,712,564 Nigerians [4]. Meanwhile, on September 30, 2021, the WHO [5] (2021b) announced a list of fifteen African countries that had effectively vaccinated 10% of their whole population; Nigeria still hadn't made the list. In May 2021, the World Health Assembly, which is the world's highest health policymaking body, announced a global aim of fully vaccinating 10% of each country's population by September 30^th^ 2021. This aim has been met by over 90% of high-income countries [5]. After two months, there hasn't been much of a difference in terms of increasing immunization to fulfill the target. According to published figures, only 1.7% of the overall population were fully vaccinated as of November 25^th^ 2021, while 1.3% were only partially vaccinated [6]. Further statistics issued by the National Primary Health Care Development Agency on December 11^th^ 2021, revealed that, as compared to states in the northern and southwest regions of the country, the southeastern states were still behind in increasing vaccination rates. Only about 90,000 and 50,000 people in each of the five southern-eastern states (Abia, Anambra, Ebonyi, Enugu, and Imo) have got the first and second doses of the COVID-19 vaccine, respectively [7].

Although specialists believe the COVID-19 vaccine is required to properly contain the pandemic, [8], as a result, public health scholars have turned to answer how many people in their countries are willing to accept the COVD-19 vaccination. And to identify the determining factors across the general population [9-14]. Previous experience with vaccines administration had often reported a low acceptance rate [15]. Earlier in 2009, poor attitudes and perceptions about vaccine reception were speculated as a significant health promotion problem [16, 17]. Public health promotion is inevitable to increase the coverage rate of these vaccines. Vaccination acceptance is a continuous necessity of concern to public health promotions, especially regarding citizens´ willingness, perceptions, and attitudes to receive newly developed vaccines such as the COVID-19 vaccine. Previous studies on readiness to receive COVID-19 vaccines have outlined such factors as beliefs about the threat of the disease [18], fears about vaccine safety, incorrect health beliefs [19, 20], and physicians´ recommendations [21-23]. Wang *et al*. [9] used the health belief model to confirm that the risk perceptions of the disease, the perceptions of vaccine safety and efficacy, attitudes and vaccination convenience, and socio-demographic variables are all determinants for accepting a pandemic vaccine. In addition, the cost, location of the development, and perceptions of the COVID-19 vaccines may be big problems for people who want to get vaccinated. These issues should be taken into account in health campaigns.

There is currently very little data in the literature that focuses on the Nigerian COVID-19 vaccine situation. While the study by Adejumo *et al*. [24] discovered that healthcare workers´ attitudes toward the COVID-19 vaccine, as well as their readiness to receive it, were both subpar, there has not been much research on the public´s willingness to receive the vaccine. Although researchers have argued that context-specific factors fuel vaccine rejection [16-18], the lack of evidence on Nigerians´ perceptions and attitudes towards COVID-19 vaccination justifies a research focus. Understanding the attitudes and perceptions of Nigerians regarding receiving COVID-19 vaccines and identifying their associated factors will be of immense importance in guiding effective interventions, strategies, or programs to help raise the vaccine acceptance rate. As a result, this study looks into the attitudes and perceptions of people in southeastern Nigeria about getting the COVID-19 vaccine, as well as the factors that make vaccines more or less popular in the five states. The findings of this study will guide health professionals, public health consultants, and local health authorities in developing and implementing an effective intervention to promote vaccine acceptance in the region, Nigeria, and globally. Since there was a link between socio-demographic characteristics and respondents' attitudes and perceptions, this study rejects the null hypothesis and accepts the alternate hypothesis. This study might be able to fill in the gaps in research on Nigerians' attitudes and perceptions about taking the COVID-19 vaccine.

## Methods

**Study design and setting:** this was a cross-sectional online survey conducted among adult residents in the five southeast geographical zones of Nigeria, which includes Anambra, Imo, Enugu, Abia, and Ebonyi, of which the majority speak Igbo and English [25]. Between July and August 2021, the researcher recruited individuals and collected data using an online platform. To keep COVID-19 from spreading between participants and researchers, the data collection was done online.

**Study population and eligibility criteria:** the people who took part in this study were adults who were (≥18 years old) and lived in the southeastern states of Nigeria. Aside from participant age, they must be from the southeastern part of Nigeria. The selection criteria also included the ability to read and understand the English language. This is because English is one of the common and official languages in these five states, as well as throughout Nigeria.

**Sample size estimation:** by calculations, the total number of participants were chosen using the formula for calculating qualitative variables in a cross-sectional survey, stated as follows [25];


$$n=\frac{Z\alpha^{2}pq}{d^{2}}$$

n= sample size (?); Zα =Is standard normal variate (at 5% type 1 error (P <0.05) it is 1.96); P=expected proportion in population based on pilot study, which is estimated at 43% of the southeastern population; q= 1-P, complementary probability of accepting the COVID-19 vaccine et d= level of precision= 0.05; therefore; sample size =


$$1.962\times 0.43\times (1-0.43)=\frac{376}{0.05^{2}}$$

The participants were recruited using the convenience sampling technique; a form of non-probability sampling method which allows participant selection based on their ease of contact/reach [26]. We only considered respondents that filled up all the sections of the questionnaire appropriately. Therefore, 334 respondents were obtained at the end of the data collection, giving a response rate of 88.8%, and hence, the researchers decided to complete the study with the data available.

**Study instrument:** a self-administered questionnaire was designed using key terms on attitudes and perceptions regarding COVID-19 vaccination from previous studies [9-14]. There were four sections to the questionnaire. The first part elicited data on socio-demographic characteristics including respondents´ age, gender, marital status, education, occupation, average household income, and ethnicity. In the second part, participants were asked about their attitudes towards COVID-19 vaccination; its efficacy, safety, and risk attribution were analysed using seven items in this area. The third part prompted questions about the perceptions of the COVID-19 vaccination, this part included seven questions with two alternative answers (“Yes” and “No”) that analysed the COVID-19 vaccine's perceptions. The content validity of the questionnaire was assessed by two epidemiologists and biostatisticians, and their recommendations were followed. Before the study began, the questionnaire was pilot tested on 20 participants who were chosen using a face-to-face purposive sampling approach for accuracy, cultural sensitivity, comprehensibility, and analysability. Cronbach's Alpha was utilized to determine its reliability. The result was 0.85, indicating that it was reliable.

**Data sources:** the adopted questionnaire was sent using a Google Form and was open to all recognized adult Nigerian residents who fit the study criteria from five southeastern states. Participants' invitations to participate in the study were distributed on social media platforms using a “one-time-only link” to the online questionnaire to prevent duplicate survey completions by the same participant. Participants were automatically directed to the survey's entrance page after clicking on this “one time only” link to the questionnaire, which provided information on the study's aims, rules of participation, and data privacy, as well as the survey's potential risks and advantages. It took roughly 10 to 15 minutes to complete the study in one sitting. The “one time only” link to the questionnaire was lost if it was not completed in one sitting. Participants could access the survey and complete it on a computer or a mobile device for convenience.

**Study analysis protocol:** a three-point Likert scale (i.e. 1 = disagree, 2 = undecided, and 3 = agree) was used to indicate the response of each item in the attitudes section. The total attitudes score was calculated by summing the raw scores of the seven items ranging from 0 to 21, with an overall greater mean score indicating more positive attitudes. Cut-off mean scores of ≥14 and <14 were used for decisions indicating positive and negative attitudes, respectively. In the perceptions' section of the instrument, each positive response by the participant was scored at 1, while negative responses were scored at 0. The total mean score was obtained by summing the raw scores of seven items ranging from 0 to 7, with higher scores above the mean indicating good perceptions towards COVID-19 vaccinations. Cut-off mean scores of ≥ 4 and < 4 were considered for decisions on good and poor perceptions, respectively. The association between socio-demographics and attitudes and perceptions was investigated using chi-square and Fisher's exact tests. All statistical tests were considered significant at the 95 percent confidence level if the p-value was less than 0.05.

The total attitude score was calculated by summing the raw scores of the seven items ranging from 0 to 21, with an overall greater mean score indicating a more positive attitude towards the COVID-19 vaccine. In the perception section of the instrument, each positive response by the participant was scored at 1, while negative responses were scored at 0. The total mean score was obtained by summing the raw scores of seven items ranging from 0 to 7, with higher scores above the mean indicating good perception towards COVID-19 vaccinations.

**Statistical data analysis:** the data was edited, sorted, and coded using a Microsoft Excel spreadsheet in a passworded computer to ensure utmost confidentiality. Data analysis was done with the aid of the Statistical Package for Social Science (SPSS) version 25 software. The data analysis included descriptive (frequency, percentage, mean, standard deviation) and inferential statistical methods (chi-square tests, Fisher's exact tests).

**Ethical issues and consent:** ethical clearance for this study was obtained from the health research ethics committee, University of Nigeria Teaching Hospital, Ituku-Mozilla Enugu, Nigeria. (With the approval number: NHREC/05/01/200B-FWA00002458-1RB00002323). All participants consented to anonymization and collation of their responses for research and publication purposes, only those that gave their consent were automatically directed to the survey page.

## Results

**Socio-demographic characteristics of the respondents:** three hundred thirty-four (334) respondents completed the online questionnaire by the end of data collection, resulting in a 100% response rate. 41.0% and 30.2% of the majorities were aged between 30 and 39 years and 18 to 29 years old, respectively. More significantly, 60.8% of the population were males. Their marital engagements showed that 59.9% were married, whereas about three-quarters (79.9%) had their diploma/ Bachelor of Science (B.Sc). In addition to this, nearly two-thirds (62.0%) of the respondents were employed. Details of the monthly earnings revealed that the majority (30.5%) earned ₦15,000- ₦30,000, followed by one-fourth (25.7%) earned between ₦30,000- ₦60,000. The table further showed the ethnic origin of the respondents, with the majority (74.0%) from Ibo, Table 1.

**Table 1:** socio-demographic characteristics of adults living in the five southeastern states of Nigeria in the year 2021

		n = 334	
Category	Options	Frequency	Percentage (%)
**Age (years)**			
	18 – 29	101	30.2
	30 – 39	137	41.0
	40 – 49	80	24.0
	50 and above	16	4.8
**Sex**			
	Male	203	60.8
	Female	131	39.2
**Marital status**			
	Single	125	37.4
	Married	200	59.9
	Divorced	-	-
	Widow/widower	5	1.5
	Others	4	1.2
**Educational qualification**			
	No formal education	3	0.9
	Primary education	1	0.3
	Secondary education	13	3.9
	Diploma/BSc	267	79.9
	MSc/PhD	50	15.0
**Occupational status**			
	Student	53	15.9
	Employed	207	62.0
	Self-employed	71	21.2
	Not working	3	0.9
	Others	-	-
**Average household income (₦)**			
	<15,000	23	6.9
	15,000 – 30,000	102	30.5
	30,000 – 60,000	86	25.7
	60,000 – 90,000	49	14.7
	>90,000	74	22.2
**Ethnicity**			
	Yoruba	25	7.5
	Igbo	244	73.0
	Hausa	4	1.2
	Others	61	18.3

Where; 1 = Disagree, 2 = Undecided and 3= Agree.

**Attitudes towards COVID-19 vaccine acceptance:** in general, there was an overall positive attitude towards the COVID-19 vaccines, with a mean score of 13.96 (SD±2.97) out of 21. This is to say that 60.2% of the total participants showed positive attitudes, as shown in Table 2. Specifically, a significant number of the respondents were certain the COVID-19 vaccines would be effective in preventing them from COVID-19 infection (53.3%) and disagreed with not receiving the COVID-19 vaccine because their bodies have natural immunity against the COVID virus (50.6%). Furthermore, nearly half agreed that they would recommend the COVID-19 vaccine to their loved ones (49.1%) and would receive the vaccine as soon as it is available (45.5%). Table 2.

**Table 2:** attitude of the adults living in the five southeastern states of Nigeria regarding receiving the COVID-19 Vaccine in the year 2021

	n = 334		
Attitude	1 (%)	2 (%)	3 (%)
I believe that COVID-19 Vaccines is not safe for my health	144(43.1)	61(18.3)	129(38.6)
I will not receive the COVID-19 vaccine because my body has natural immunity against the COVID -19 virus	169(50.6)	57(17.1)	108(32.3)
Would receive COVID -19 vaccination as soon as the vaccine is available	134(40.1)	48(14.4)	152(45.5)
I will recommend COVID-19 vaccine to my loved ones	164(49.1)	50(15.0)	120(35.9)
COVD-19 vaccine will quickly stop the pandemic	170(51.0)	60(17.9)	104(31.1)
I am sure the COVID-19 vaccines will be effective in preventing me from COVID-19 infection	99(29.6)	57(17.1)	178(53.3)
I will only receive COVID-19 vaccine if it is given for free	84(25.1)	66(19.8)	184(55.1)
**The overall mean score**			**13.96±2.97**
**The overall attitude of the respondents**	**Frequency**		**Percentage**
Positive	201		60.2
Negative	133		39.8

**Perceptions of COVID-19 vaccine acceptance:** overall, there were poor perceptions of the COVID-19 vaccination, with a summed mean score of 3.30 (SD ±1.17) out of 7. As shown in Table 3, only 47.1% of all respondents had positive perceptions of vaccine acceptance, while 53.0% had negative perceptions. Given the widespread vaccine acceptance, the majority of people still doubted the existence of the COVID-19 virus (63.8%) and did not believe they were in danger of infection (58.4%). Children should be vaccinated, and the COVID-19 vaccine will protect them against the COVID-19 virus for the rest of their lives, according to 60.8% and 58.8% of respondents respectively Table 3.

**Table 3:** perception of the adults living in the five southeastern states of Nigeria regarding receiving the COVID-19 Vaccine in the year 2021

		n = 334	
Perception		Yes (%)	No (%)
A vaccine is important to end the COVID-19 pandemic?		188(56.3)	146(43.7)
Will it be possible to produce safe and effective vaccines?		213(63.8)	121(36.2)
Would you pay to be vaccinated if is not given for free?		174(52.1)	160(47.9)
Should children be vaccinated too?		203(60.8)	131(39.2)
Do you think that the Covid-19 Vaccine will give you lifelong protection against the COVID-19 virus		196(58.7)	138(41.3)
Do you believe the COVID-19 virus is real?		121(36.2)	213(63.8)
Do you feel at risk of getting infected with the COVID-19 virus?		139(41.6)	195(58.4)
**The overall mean score**			**3.30±1.17**
**The overall perception of the respondents**	**Frequency**		**Percentage**
Good	157		47
Poor	177		53

**Association between Socio-demographic characteristics with attitudes and perceptions:** there was a statistically significant association between socio-demographic with attitudes and perceptions towards COVID-19 vaccine acceptance among adults in five south-eastern states of Nigeria (Table 4). Only the respondents' gender (p< 0.001) was shown to be statistically connected with their attitudes towards COVID-19 vaccine acceptance. Table 4. There was also a strong statistically significant association between respondents´ perceptions of the COVID-19 acceptability and their age (p< 0.001), gender (P < 0.001), level of education (P< 0.001), and marital status (P< 0.001). Table 4.

**Table 4:** association of socio-demographic variables with the attitude and perception of adults living in five Southeastern Nigerian regarding receiving COVID-19 vaccines

	Attitude					Perception				
Socio-demographic variables	Negative	Positive	Total	%	P-value	Good	Poor	Total	%	P-value
**Age (years**)					0.774					0.001
18 – 29	36	65	101	30.2		57	44	101	30.2	
30 – 39	55	82	137	41		73	64	137	41	
40 – 49	36	44	80	24		37	43	80	24	
50 and above	6	10	16	4.8		10	6	16	4.8	
**Sex**					0.001					0.001
Male	11	81	203	60.8		110	93	203	60.8	
Female	122	120	131	39.2		67	64	131	39.2	
**Marital status**					0.793					0.001
Single	46	79	125	37.4		68	57	125	37.4	
Married	82	118	200	59.9		103	97	200	59.9	
Widow/widower	3	2	5	1.5		4	1	5	1.5	
Others	2	2	4	1.2		2	2	4	1.2	
**Educational qualification**					0.810					0.001
No formal education	2	1	3	0.9		2	1	3	0.9	
Primary education	1	-	1	0.3		1	-	1	0.3	
Secondary education	5	8	13	3.9		8	5	13	3.9	
Diploma/BSc	104	163	267	79.9		143	124	267	79.9	
MSc/PhD	21	29	50	15		23	27	50	15	

**Association between attitudes and perceptions towards COVID-19 vaccine acceptance:** there was a statistically significant association between attitude and perception regarding receiving the COVID-19 vaccines among adult respondents in the southeastern part of Nigeria (p<0.001). Table 5. This reveals that participants who had a positive attitude also possessed good perceptions.

**Table 5:** chi-square test of association of attitude with the perception of adults from five south-eastern states of Nigeria towards receiving COVID-19 vaccines

	Attitude				
Perception	Negative	Positive	Total	Chi-square	P-value
Poor	93	84	177	25.432	< 0.001
Good	40	117	157		
Total	133	201	334		

Level of significance at < 0.05

## Discussion

This present study assessed the attitudes and perceptions of Nigerians regarding receiving the COVID-19 vaccination. The study's finding showed that the majority of young people (60.2%) in five Southeastern Nigerian states expressed positive attitudes about receiving the vaccination, with gender being the sole significant factor and more females possessing positive attitudes than males. However, their poor perception of the COVID-19 vaccine acceptance (53.0%) was linked with some of their socio-demographic factors, such as gender, age, education, and marital status. Furthermore, the relationship between the respondents' attitudes and perceptions was found to be significant.

This study revealed overall positive attitudes toward receiving the COVID-19 vaccination, which contrasts a Shekhar *et al*., [27] survey among HCWs in the United States, that found negative attitudes toward receiving the COVID-19 vaccine during its early vaccine development in September 2020. However, these findings are similar to a survey conducted in the Indian population one year following the vaccine's development, where half of the respondents had positive attitudes regarding COVID-19 acceptance [28]. Another published study conducted in two Nigerian universities found that the respondents had positive attitudes [29]. These positive attitudes one year after the vaccine development might be due to an increase in awareness and health education towards the vaccination´s benefits in the fight against the pandemic. In this study, being male was more strongly connected with negative attitudes toward COVID-19 acceptability, which contradicts a recent article from Northern Nigeria [30] and Bangladesh [31] that claimed being a man is a factor of COVID-19 vaccine acceptance. Further contrasting findings were observed in an Ishimaru *et al*.study [32], in which the researchers observed that women were less willing to receive vaccination than men. In comparison to this study, a poll in Bangladesh found that female respondents were more willing to receive the COVID-19 vaccine than male respondents [33]. These data could aid in identifying target populations for increasing COVID-19 vaccination awareness, especially among women in Nigeria.

Although a higher number of respondents had positive attitudes towards vaccination acceptance, their perspective was nevertheless negative in this survey. In a similar study conducted in Bangladesh, more than half of the respondents had negative perceptions towards getting COVID-19 shots [34], this was due to the many falsehoods that greatly impacted people's perceptions of getting the COVID-19 vaccine. Whereas one study in Nigeria found that health workers have a positive view of COVID-19 [35], this could be attributable to a high level of knowledge and information about vaccine safety among the said population.

In this study, 38.6% of participants thought the vaccine would have some negative effects and hence was not safe for them, which is similar to findings from studies in the United States [36] and Bangladesh [33, 34]. In a study conducted in China, more than half of the respondents said they are worried about the vaccine's side effects and would delay the COVID-19 vaccination until the vaccine's safety is confirmed [37], reflecting their uncertainty regarding vaccine safety, even though they believe it´s important to stop the pandemic. This may be related to misleading information spread through the media and social media that encourages vaccine resistance and rejection [38]. This insight was supported by the findings of Harrison and Wu's [20] and Puri *et al*. [21] which highlighted that vaccine safety worries and inaccurate health beliefs are important predictors of vaccination willingness. The majority of people in this survey still deny the virus' existence and feel that their natural immunity will help them fight it, so they are not at risk of infection. This finding is similar to a study in one of the villages in India [28], respondents doubted the safety of the vaccine, placed more trust in their bodies' immune systems than in the vaccine, and were concerned about the vaccine's long-term effects. This may be possible due to conspiracy theories and religious views about the vaccine and the COVID-19 virus. These findings indicate that there is a significant need for additional and strategic public awareness and educational programs that will provide accurate and reliable information to the public regarding the COVID-19 vaccine benefits. This can be achieved through credible and respected national and community health promotion platforms. This idea was supported by Schmidt *et al*. [39] who suggested that generating community-specific communication materials could help to address misconceptions about COVID-19 vaccination acceptance.

It's significant to observe that in this study, various socio-demographic characteristics such as age, gender, post-secondary educational qualification, and marital status were statistically connected with the perceptions of the COVID-19 vaccine acceptance in Nigeria's five south-eastern states. This is similar to a published article in 2021 among Bangladesh's general population [31], and Sarah *et al*. [40], which reported that socio-demographic factors such as age, gender, education, and employment influence COVID-19 risk perceptions and COVID-19 acceptability. In this study, they are more likely to get the vaccine if they haven't been married, which is in contrast to a survey of 1,725 Bangladesh adults that found those who haven't been married were less likely to get the COVID-19 vaccine. [41]. We also noticed in this survey that elderly people are more inclined to accept the vaccine than younger people, which was validated in a survey among health science students in Northwest Nigeria, where the older respondents expressed more willingness to accept the vaccine. [42]. In this study, the post-secondary education level was found to influence the perceptions of COVID-19 vaccine acceptance. This is similar to a study conducted among adults in the Ontario community [31, 43], which found that people without a bachelor's degree are less likely to accept the vaccine. This could be due to a lack of accurate information regarding COVID-19 among those without a post-secondary education. Most people without a bachelor's degree in the southeastern area of Nigeria rarely display an interest in health-related topics, which may explain why they lack basic health education and information about COVID-19. As a result, health education should be spread to their businesses and places of work.

Nonetheless, we identified a link between perceptions and attitudes, with participants who have positive attitudes also having good perceptions. This link was found in a survey of 1500 adults in the United Kingdom, China, and public university students in Bangladesh [9, 13, 43]. In our study, this association was more common among those with a post-secondary degree who have not yet married, which could be due to their higher level of education that aids them in having a better understanding of COVID-19 transmission and intervention. In this research, for example, most respondents who believe COVID-19 vaccination will immediately stop the pandemic and protect them from infection had a greater degree of confidence, which is likely why they will recommend the vaccine to their loved ones, compared to those who disagree. Therefore, a health education intervention program could help increase awareness of the COVID-19 vaccine benefit in controlling the pandemic, especially among those without a post-secondary education.

**Limitations:** some limitations in this study´s generalizability are worthy of consideration in the interpretation of the study findings. Firstly, the cross-sectional study design limited any ability to make a causal inference concerning the association between attitudes, perceptions, and the readiness to receive the COVID-19 vaccines. Secondly, due to the online self-reporting method used by the study, there may have been an increased tendency for social desirability bias in the responses. Thirdly, there are concerns about the sampling frame, as only a representative sample of the Nigerian population´s southeast region was included in this study. Lastly, the sample size for this study was not up to the estimated sample due to unwillingness to participate in anything related to COVID-19. Further studies should consider giving incentives to the participants to help increase the sample size. Considering the limitations identified, the outcome of this research will help experts provide useful information and insight on how to strategically engage the public to accept the COVID-19 vaccine, as well as support for future vaccine acceptance research. As a result, a lot of research that includes the whole Nigerian community is needed to help make better policy decisions about this subject.

## Conclusion

This online cross-sectional survey revealed positive attitudes and negative perceptions towards the acceptance of the COVID-19 vaccine in Nigeria's five southeastern states. Age, gender, educational level, and marital status were the main determinants of vaccine acceptability. It also demonstrated a link between respondents' attitudes and perceptions, which shows that those who had positive attitudes also had good perceptions. Therefore, aside from a recent study that explored public health promotion and intervention initiatives in pandemics like this, national and community-based intervention initiatives that target those without a formal education, young adults, and married people should be deliberately strengthened. To increase engagement and involvement, celebrities, community leaders, and religious leaders could be included. Personal-centered public health programs to promote proper awareness of COVID-19, as well as their attitudes and perceptions of vaccine acceptance, are still required and encouraged.

### What is known about this topic

- Experts´ projections suggest that the COVID-19 vaccine is necessary for successfully controlling the pandemic;
- Previous experience with vaccines administration had often reported a low acceptance rate due to the interference of some factors such as beliefs about the threat of the disease, fears about vaccine safety, incorrect health beliefs, and physicians´ recommendation.

### What this study adds

- The majority of younger age groups, as well as those who have never completed post-secondary education, do not believe the COVID-19 virus exists, according to the findings of this study. As such, these groups mostly do not consider themselves in danger of becoming infected, which is why they will not receive the vaccine;
- To make the COVID-19 vaccine benefit more obvious and acceptable to this population, additional emphasis on community and health education should be placed in commercial places and churches where these people are most likely to be found;
- This study also reveals that respondents that are unmarried, older, with post-secondary educational qualifications, and mostly female expressed a positive willingness to accept the vaccine.

## References

[1] World Health Organization (WHO) Ten threats to global health in 2019.

[2] World Health Organization (WHO) WHO Coronavirus (COVID-19) Dashboard.

[3] NCDC COVID-19 NIGERIA.

[4] World Population Review Nigeria Population 2022 (Live).

[5] Our World in Data Coronavirus Pandemic (COVID-19).

[6] NPHCDA Welcome Message from the ED/CEO.

[7] Corey L, Mascola JR, Fauci AS, Collins FS (2020). A strategic approach to COVID-19 vaccine R&D. Science.

[8] Wang J, Jing R, Lai X, Zhang H, Lyu Y, Knoll MD (2020). Acceptance of COVID-19 Vaccination during the COVID-19 Pandemic in China. Vaccines (Basel).

[9] Graffigna G, Palamenghi L, Boccia S, Barello S (2020). Relationship between Citizens´ Health Engagement and Intention to Take the COVID-19 Vaccine in Italy: A Mediation Analysis. Vaccines (Basel).

[10] Pogue K, Jensen JL, Stancil CK, Ferguson DG, Hughes SJ, Mello EJ (2020). Influences on Attitudes Regarding Potential COVID-19 Vaccination in the United States. Vaccines (Basel).

[11] Head KJ, Kasting ML, Sturm LA, Hartsock JA, Zimet GD (2020). A National Survey Assessing SARS-CoV-2 Vaccination Intentions: Implications for Future Public Health Communication Efforts. Science Communication.

[12] Sherman SM, Smith LE, Sim J, Amlôt R, Cutts M, Dasch H (2021). COVID-19 vaccination intention in the UK: results from the COVID-19 vaccination acceptability study (CoVAccS), a nationally representative cross-sectional survey. Hum Vaccin Immunother.

[13] Reiter PL, Pennell ML, Katz ML (2020). Acceptability of a COVID-19 vaccine among adults in the United States: How many people would get vaccinated?. Vaccine.

[14] Biasio LR, Bonaccorsi G, Lorini C, Pecorelli S (2021). Assessing COVID-19 vaccine literacy: a preliminary online survey. Hum Vaccin Immunother.

[15] Eastwood K, Durrheim DN, Jones A, Butler M (2010). Acceptance of pandemic (H1N1) 2009 influenza vaccination by the Australian public. Med J Aust.

[16] World Health Organization (WHO) Behavioural considerations for acceptance and uptake of COVID-19 vaccines: WHO technical advisory group on behavioural insights and sciences for health, meeting report, 15 October 2020.

[17] MacDonald NE (2015). Vaccine hesitancy: Definition, scope and determinants. Vaccine.

[18] Larson HJ, Jarrett C, Eckersberger E, Smith DMD, Paterson P (2014). Understanding vaccine hesitancy around vaccines and vaccination from a global perspective: A systematic review of published literature, 2007-2012. Vaccine.

[19] Harrison EA, Wu JW (2020). Vaccine confidence in the time of COVID-19. Eur J Epidemiol.

[20] Puri N, Coomes EA, Haghbayan H, Gunaratne K (2020). Social media and vaccine hesitancy: new updates for the era of COVID-19 and globalized infectious diseases. Hum Vaccin Immunother.

[21] Moss JL, Reiter PL, Rimer BK, Brewer NT (2016). Collaborative patient-provider communication and uptake of adolescent vaccines. Soc Sci Med.

[22] Gilkey MB, Calo WA, Moss JL, Shah PD, Marciniak MW, Brewer NT (2016). Provider communication and HPV vaccination: The impact of recommendation quality. Vaccine.

[23] Lu PJ, Srivastav A, Amaya A, Dever JA, Roycroft J, Kurtz MS (2018). Association of provider recommendation and offer and influenza vaccination among adults aged ≥18 years-United States. Vaccine.

[24] Charan J, Biswas T (2013). How to Calculate Sample Size for Different Study Designs in Medical Research?. Indian Journal of Psychological Medicine.

[25] Lavrakas P Encyclopedia of Survey Research Methods 2008. 2455 Teller Road, Thousand Oaks California 91320 United States of America. Sage Publications, Inc.

[26] Shekhar R, Sheikh AB, Upadhyay S, Singh M, Kottewar S, Mir H (2021). COVID-19 Vaccine Acceptance among Health Care Workers in the United States. Vaccines (Basel).

[27] Danabal KGM, Magesh SS, Saravanan S, Gopichandran V (2021). Attitude towards COVID 19 vaccines and vaccine hesitancy in urban and rural communities in Tamil Nadu, India-a community based survey. BMC Health Serv Res.

[28] Adetayo AJ, Sanni BA, Aborisade MO (2021). COVID-19 Vaccine Knowledge, Attitude, and Acceptance among Students in Selected Universities in Nigeria: DSAHMJ.

[29] Lawal N, Bello MB, Yakubu Y, Ibrahim AM, Rabiu SA (2022). Appraisal of the knowledge, attitude, perception and practices among northern Nigerians in the wake of the COVID-19 outbreak. Future Science OA.

[30] Mahmud S, Mohsin M, Khan IA, Mian AU, Zaman MA (2021). Knowledge, beliefs, attitudes and perceived risk about COVID-19 vaccine and determinants of COVID-19 vaccine acceptance in Bangladesh. PLoS One.

[31] Ishimaru T, Okawara M, Ando H, Hino A, Nagata T, Tateishi S (2021). Gender differences in the determinants of willingness to get the COVID-19 vaccine among the working-age population in Japan. Hum Vaccin Immunother.

[32] Islam MdS, Siddique AB, Akter R, Tasnim R, Sujan MdSH, Ward PR (2021). Knowledge, attitudes and perceptions towards COVID-19 vaccinations: a cross-sectional community survey in Bangladesh. BMC Public Health.

[33] Bari MdS, Hossain MdJ, Ahmmed F, Sarker MdMR, Khandokar L, Chaithy AP (2021). Knowledge, Perception, and Willingness towards Immunization among Bangladeshi Population during COVID-19 Vaccine Rolling Period. Vaccines (Basel).

[34] Adejumo OA, Ogundele OA, Madubuko CR, Oluwafemi RO, Okoye OC, Okonkwo KC (2021). Perceptions of the COVID-19 vaccine and willingness to receive vaccination among health workers in Nigeria. Osong Public Health Res Perspect.

[35] Callaghan T, Moghtaderi A, Lueck JA, Hotez P, Strych U, Dor A (2021). Correlates and disparities of intention to vaccinate against COVID-19. Soc Sci Med.

[36] Zhou Q, Tian T, Ni J, Zhao X, Li H, Yang Y (2021). COVID-19 Vaccination Acceptance in China after It Becomes Available: A Cross-Sectional Study. Vaccines (Basel).

[37] Islam MS, Kamal A-HM, Kabir A, Southern DL, Khan SH, Hasan SMM (2021). COVID-19 vaccine rumors and conspiracy theories: The need for cognitive inoculation against misinformation to improve vaccine adherence. PLoS One.

[38] Schmidt T, Cloete A, Davids A, Makola L, Zondi N, Jantjies M (2020). Myths, misconceptions, othering and stigmatizing responses to Covid-19 in South Africa: A rapid qualitative assessment. PLoS One.

[39] Dryhurst S, Schneider CR, Kerr J, Freeman ALJ, Recchia G, van der Bles AM (2020). Risk perceptions of COVID-19 around the world. Journal of Risk Research.

[40] Kamal A-HM, Sarkar T, Khan MM, Roy SK, Khan SH, Hasan SMM (2021). Factors Affecting Willingness to Receive COVID-19 Vaccine Among Adults: A Cross-sectional Study in Bangladesh. Journal of Health Management.

[41] Mustapha M, Lawal BK, Sha´aban A, Jatau AI, Wada AS, Bala AA (2021). Factors associated with acceptance of COVID-19 vaccine among University health sciences students in Northwest Nigeria. PLoS One.

[42] Syan SK, Gohari MR, Levitt EE, Belisario K, Gillard J, DeJesus J (2021). COVID-19 Vaccine Perceptions and Differences by Sex, Age, and Education in 1,367 Community Adults in Ontario. Front Public Health.

[43] Hossain ME, Islam MS, Ghose TK, Jahan H, Chakrobortty S, Hossen MdS (2021). COVID-19 vaccine acceptability among public university students in Bangladesh: Highlighting knowledge, perceptions, and attitude. Hum Vaccin Immunother.

